# The maize ALDH protein superfamily: linking structural features to functional specificities

**DOI:** 10.1186/1472-6807-10-43

**Published:** 2010-12-29

**Authors:** Jose C Jimenez-Lopez, Emma W Gachomo, Manfredo J Seufferheld, Simeon O Kotchoni

**Affiliations:** 1 Department of Biochemistry, Cell and Molecular Biology of Plants; Estacion Experimental del Zaidin (EEZ), Consejo Superior de Investigaciones Cientificas (CSIC), Profesor Albareda 1, E-18008, Granada, Spain; 2 Department of Botany and Plant Pathology, Purdue University, Lilly Hall, 915 West State Street, West Lafayette, IN 47907, USA; 3 Department of Crop Science, University of Illinois U-C, Urbana-Champaign, Illinois, USA; 4 Department of Agronomy, Purdue University, Lilly Hall, 915 West State Street, West Lafayette, IN 47907, USA; 5 Department of Biological Sciences, Purdue University, West Lafayette, IN 47907, USA

## Abstract

**Background:**

The completion of maize genome sequencing has resulted in the identification of a large number of uncharacterized genes. Gene annotation and functional characterization of gene products are important to uncover novel protein functionality.

**Results:**

In this paper, we identify, and annotate members of all the maize aldehyde dehydrogenase (ALDH) gene superfamily according to the revised nomenclature criteria developed by ALDH Gene Nomenclature Committee (AGNC). The maize genome contains 24 unique *ALDH *sequences encoding members of ten ALDH protein families including the previously identified male fertility restoration *RF2A *gene, which encodes a member of mitochondrial class 2 ALDHs. Using computational modeling analysis we report here the identification, the physico-chemical properties, and the amino acid residue analysis of a novel tunnel like cavity exclusively found in the maize sterility restorer protein, RF2A/ALDH2B2 by which this protein is suggested to bind variably long chain molecular ligands and/or potentially harmful molecules.

**Conclusions:**

Our finding indicates that maize ALDH superfamily is the most expanded of plant *ALDHs *ever characterized, and the mitochondrial maize RF2A/ALDH2B2 is the only plant ALDH that harbors a newly defined pocket/cavity with suggested functional specificity.

## Background

Endogenous aldehyde molecules are intermediates/by-products of several fundamental metabolic pathways [1], and are also produced in response to environmental stresses including salinity, dehydration, desiccation, cold, and heat shock [2,3]. Although indispensable to biological processes, they are however toxic in excessive physiological concentrations [4]. The damaging effects of aldehydes and derivatives of aldehyde molecules, which include cytotoxicity, mutagenicity, and carcinogenicity, have been well studied in human, bacteria and fungi [4,5]. Therefore, cellular levels of aldehydes must be regulated to ensure normal developmental growth processes.

Aldehyde dehydrogenases (ALDHs) constitute a large family of NAD(P)^+^-dependent enzymes that catalyze the irreversible oxidation of a wide range of reactive aldehydes to their corresponding carboxylic acids [2]. In additions, ALDHs have been shown to indirectly detoxify cellular ROS and reduced the effect of lipid peroxidation mediated cellular toxicity under drought and salt stress [6]. ALDHs are found in both prokaryotes and eukaryotes. With the genome of more organisms being fully sequenced, the numbers of ALDH genes identified have lately increased [1,4,7,8]. However, relatively few studies have been conducted on the corresponding plant enzymes and specifically on maize-ALDHs.

The availability of recently sequenced maize genome [9] has provided an avenue for gene discovery, functional and comparative genomics studies. This avails a basis for a close investigation into phylogenetic analysis and structural features of all maize ALDHs compared to other well characterized plant ALDHs. Criteria for unified ALDH nomenclature have been well established by the ALDH Gene Nomenclature Committee (AGNC) [10]. Based upon these criteria, protein sequences with more than 40% identity to a previously identified ALDH sequence represent a family, and sequences with more than 60% identity within the ALDH family represent a protein subfamily [10]. We present here a revised and unified nomenclature for the maize ALDH superfamily according to AGNC criteria [10].

Some plants express mitochondrial genes that cause cytoplasmic male sterility (CMS), however, nuclear genes that disrupt the accumulation of the corresponding mitochondrial gene products can restore fertility to such plants. CMS is a maternally inherited trait that is observed in more than 150 higher plant species including maize. The hybrid vigor in higher plants depends on the use of CMS, which is characterized by the absence of functional pollen. CMS is a useful system for commercial F1 hybrid breeding programs. In maize the male sterility is caused by a Texas cytoplasm-specific mitochondrial gene (CMS-T), *T*-*URF13 *that encodes a 13 kDa URF13 protein [11]. The dominant alleles for fertility restoration (*RF*) *RF1 *and *RF2 *(also known as *RF2A*) have been shown to work together to restore the URF13-mediated sterility [12,13]. Although many mitochondrial genes associated with CMS have been characterized, the identification and characterization of *RF *genes has proven elusive, and only the maize *RF2A*, which encodes a mitochondrial ALDH, ALDH2B2, is the most well characterized *RF *gene so far [12,13]. Up to date, the mechanism by which URF13 causes male sterility in maize is not known, and the functional features of male sterility restorer, RF2A/ALDH2B2, is completely unknown. In addition, the maize line carrying Texas male sterile cytoplasm is highly susceptible to southern corn leaf blight, one of the worst plant disease caused by *Cochliobolus heterostrophus *race T, which produces a polyketide T-toxin, a determinant of the fungal virulence. Using computational modeling, we have identified a novel tunnel like shape ligand binding cavity in the male sterility restorer, RF2A/ALDH2B2 protein of maize. Computational modeling is a powerful tool to predict protein structures, functions and protein-protein or protein-ligand interactions. Domain organization of proteins is an intrinsic element of protein structure and functionality. Therefore, understanding the domain organizations of proteins is a prerequisite to efficiently manipulating and predicting the folding structure mediating functionality. The specific biochemical pathway(s) of plant ALDHs is an area of considerable interest. To better understand the roles of RF2A/ALDH2B2, we explore in detail the structural features of the maize RF2A/ALDH2B2 tunnel like cavity and discuss here it functional relevance compared to other members of maize ALDH families.

## Results

### The maize ALDH gene superfamily: revised nomenclature and phylogenetic analysis

**Figure 1:**
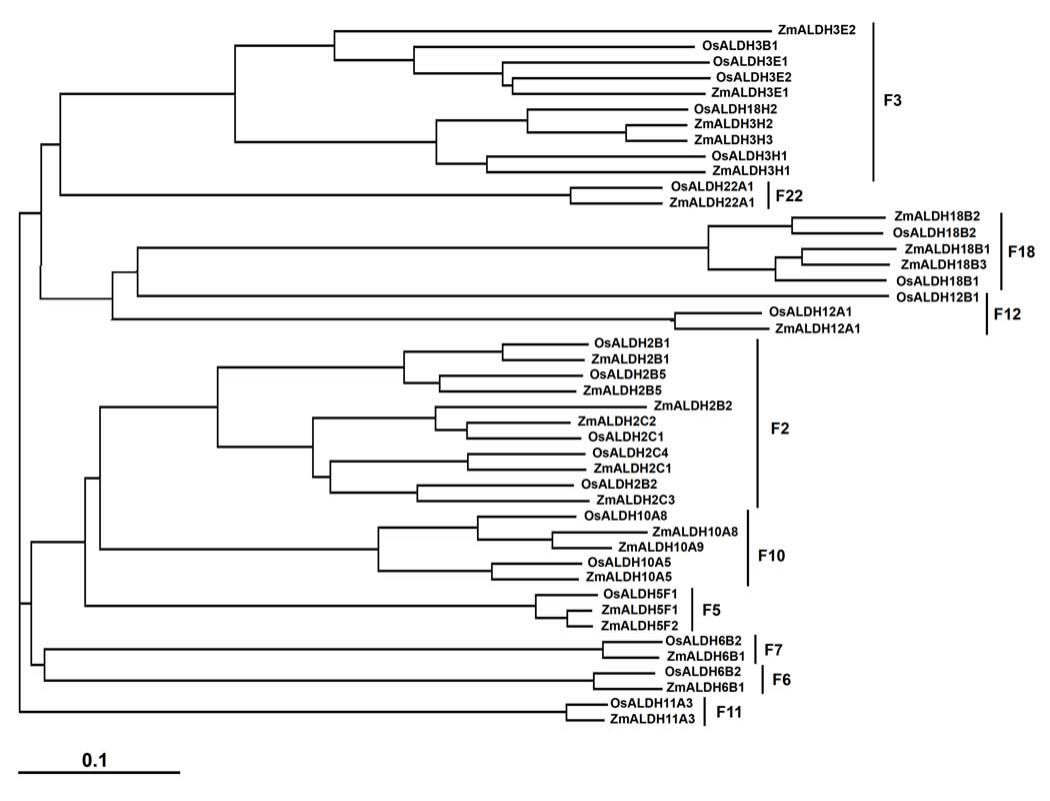
**Phylogenetic relationship of maize and rice ALDHs**. Neighbor-Joining (NJ) method was used to perform a phylogenetic analysis and respective ALDH families were indicated.

**Figure 2:**
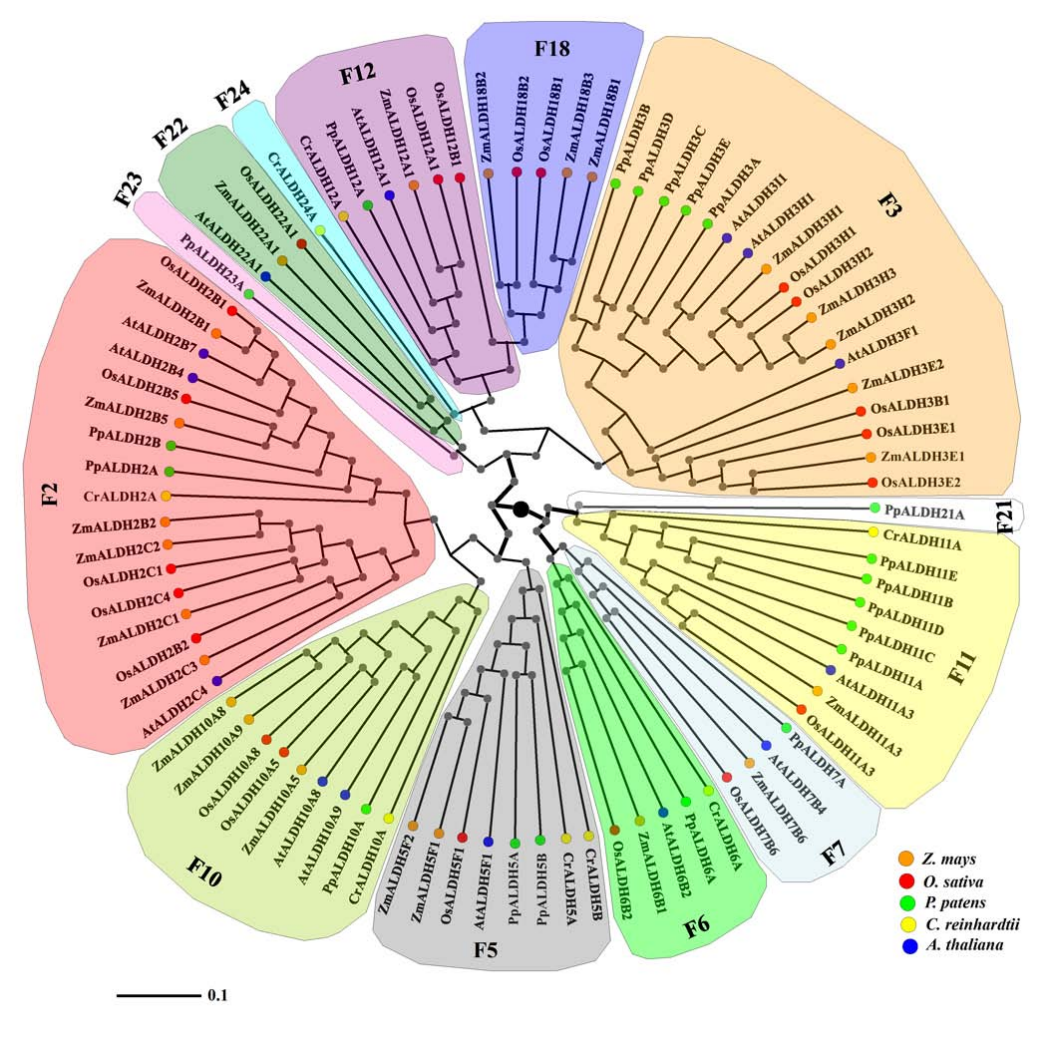
**Phylogenetic analysis of maize ALDHs with other well characterized plant ALDHs**. Neighbor-Joining (NJ) method was used to perform a phylogenetic analysis of *Z. mays *(orange), *O. sativa *(red), *A. thaliana *(blue), *P. patens *(green), and *C. reinhardtii *(yellow) deduced ALDH protein sequences. Members of respective ALDH families are depicted in a specific background color.

The release of maize genome sequence provides a powerful tool for identification and functional characterization of genes. Here, we have searched the entire maize genome [9] and assigned ALDH nomenclature to identified maize genes based on sequence similarity of deduced amino acids to previously characterized ALDH genes (Table 1). To ensure the accuracy of the sequences used in the maize *ALDH *gene superfamily identification, we used ALDH conserved motifs, ALDH active sites and ALDH defined family criteria (as detailed in the Materials and Methods) and the Arabidopsis *ALDH *gene superfamily [7] as database search queries. We verified all annotated maize ALDH open reading frames (ORFs) by comparing them to the cDNA and EST sequences. The search resulted in the identification of 24 unique *ALDH *sequences encoding members of ten ALDH protein families (Table 1), two of which (family 2: ALDH2B1, ALDH2B2; family 11: ALDH11A3) have been previously identified [14]. Compared to other well characterized plant ALDHs, maize-*ALDH *gene superfamily is the most expanded with 24 vs. 21 genes in rice [15]; 20 genes in moss [8]; 8 genes in algae [8]; and 14 genes in *Arabidopsis thaliana *[7]. Five (ALDH2: 6genes; ALDH3: 5 genes; ALDH5: 2 genes; ALDH10: 3 genes; ALDH18: 3 genes) out of the ten ALDH families are represented by multiple *ALDH *gene members (Table 1), while the remaining five families (6; 7; 11; 12; 22) are represented by a single *ALDH *gene copy (Table 1). As expected, the phylogenetic analysis showed that *Z. mays *ALDH sequences are more closely related to *Oryza sativa *(Figure 1) and *A. thaliana*, than to *P. patens *and *C. reinhardtii *ALDHs (Figure 2), with ADLH23 and ALDH24 found only in *P. patens *and *C. reinhardtii *genome respectively, and *C. reinhardtii *lacking the ALDH3 and ALDH7 gene families (Figure 2). A phylogenetic analysis of maize ALDH sequences with other putative plant ALDHs revealed that plant ALDHs are split into four clades and maize-ALDHs share common core plant ALDH families (ALDH2, ALDH3, ALDH5, ALDH6, ALDH7, ALDH10, ALDH11, ALDH12 and ALDH22) (Table 2; Figure 2).

**Table 1:** The maize ALDH protein superfamily: revised and unified nomenclature

ALDH Family	Revised Annotation	Accession Number	Molecular Function	Subcellular Localization
Family2	ZmALDH2B1	AC189099.3	Aldehyde dehydrogenase	Mitochondria
	ZmALDH2B2	AC191038.4	Aldehyde dehydrogenase/RF2A	Mitochondria
	ZmALDH2B5	AC182825.4	Aldehyde dehydrogenase/RF2B	Mitochondria
	ZmALDH2C1	AC203907.3	Aldehyde dehydrogenase/RF2C	Cytosol
	ZmALDH2C2	AC212213.3	Aldehyde dehydrogenase/RF2D	Cytosol
	ZmALDH2C3	AC200510.3	Aldehyde dehydrogenase	Cytosol
				
Family 3	ZmALDH3E1	AC194279.3	Aldehyde dehydrogenase	Chloroplast
	ZmALDH3E2	AC206699.3	Aldehyde dehydrogenase	Chloroplast
	ZmALDH3H1	AC196114.2	Aldehyde dehydrogenase [NAD(P)+]	Chloroplast
	ZmALDH3H2	AC204269.3	Aldehyde dehydrogenase [NAD(P)+]	Chloroplast
	ZmALDH3H3	AC177866.2	Aldehyde dehydrogenase [NAD(P)+]	Endoplasmic reticulum
				
Family 5	ZmALDH5F1	AC191786.3	Succinate semialdehyde dehydrogenase	Mitochondria
	ZmALDH5F2	AC196023.3	Succinate semialdehyde dehydrogenase	Mitochondria
				
Family 6	ZmALDH6B1	AC191354.3	Methylmalonate semi-aldehyde dehydrogenase	Mitochondria
				
Family 7	ZmALDH7B6	AC196479.3	Antiquitin	Cytosol
				
Family 10	ZmALDH10A5	AC203433.3	Betaine-aldehyde dehydrogenase	Peroxisome
	ZmALDH10A8	AC193454.3	Betaine-aldehyde dehydrogenase	Peroxisome
	ZmALDH10A9	AC205791.3	Betaine-aldehyde dehydrogenase	Peroxisome
				
Family 11	ZmALDH11A3	AC219083.3	NADH-dependent glyceraldehyde-3-phosphate dehydrogenase	Cytosol
				
Family 12	ZmALDH12A1	AC211544.3	Delta-1-pyrroline-5-carboxylate dehydrogenase	Mitochondria
				
Family 18	ZmALDH18B1	AC231617.2	Delta-1-pyrroline-5-carboxylate synthetase	Cytosol
	ZmALDH18B2	AC208465.3	Delta-1-pyrroline-5-carboxylate synthetase	Cytosol
	ZmALDH18B3	AC203754.4	Delta-1-pyrroline-5-carboxylate synthetase	Cytosol
				
Family 22	ZmALDH22A1	AC212124.5	Aldehyde dehydrogenase	Secretory pathway/endomembrane compartment

**Table 2:** Comparative study of the ALDH gene families identified in various organisms

Organism	ALDH family																							
	
	1	2	3	4	5	6	7	8	9	10	11	12	13	14	15	16	17	18	19	20	21	22	23	24
*Z. mays*	-	+	+	-	+	+	+	-	-	+	+	+	-	-	-	-	-	+	-	-	-	+	-	-

*O. sativa*	-	+	+	-	+	+	+	-	-	+	+	+	-	-	-	-	-	+	-	-	-	+	-	-
*P. patens*	-	+	+	-	+	+	+	-	-	+	+	+	-	-	-	-	-	-	-	-	+	-	+	-
*A. thaliana*	-	+	+	-	+	+	+	-	-	+	+	+	-	-	-	-	-	-	-	-	-	+	-	-
*C. reinhardtii*	-	+	-	-	+	+	-	-	-	+	+	+	-	-	-	-	-	-	-	-	-	-	-	+
*Human*	+	+	+	+	+	+	+	+	+	-	-	-	-	-	-	-	-	+	-	-	-	-	-	-
*Fungi*	+	-	-	+	+	-	-	-	-	+	-	-	-	+	+	+	-	+	-	-	-	-	-	-

Presence (+) or absence (-) of *ALDH *gene family is depicted in each indicated organism.

### Structural characterization of maize sterility restorer, RF2A, a member of class 2 ALDHs

**Figure 3:**
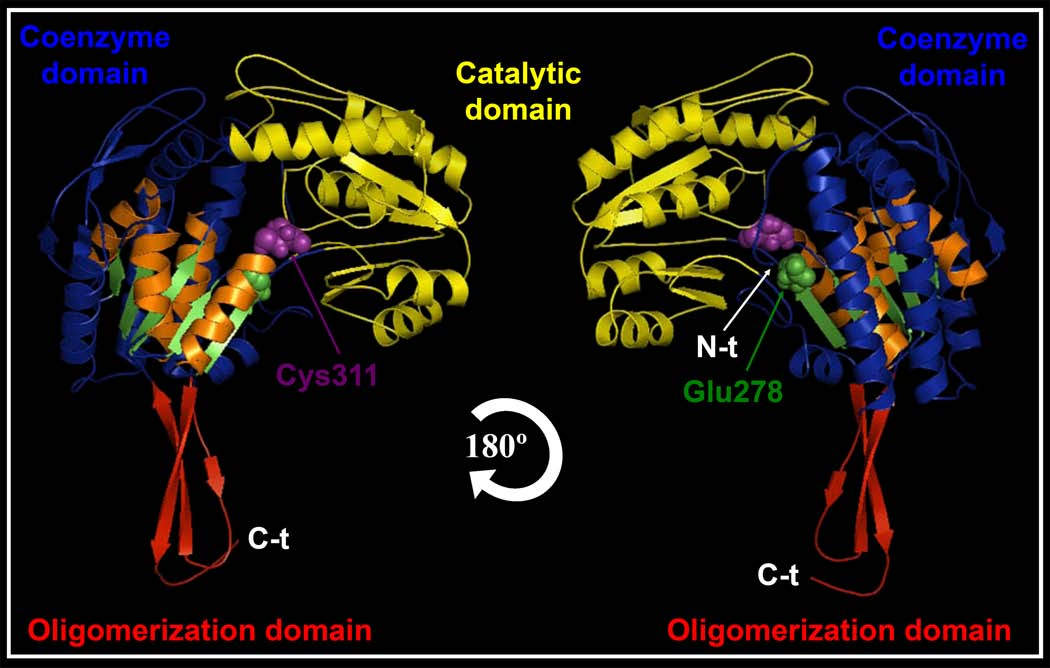
**Domain organization of maize ALDH2B2/RF2A monomer**. ZmALDH2B2/RF2A structural subunit is color coded to distinguish the oligomerization domain (red), coenzyme domain (blue) and the catalytic domain (yellow). Part of the common strands and helices in the Rossmann type fold that integrate the NAD(P)^+ ^coenzyme pocket is highlighted in orange and green color. The Cys311 (purple) and Glu278 (green) amino acids, which interact with the aldehyde substrate and drive the ALDH enzymatic reaction is depicted in the spherical shapes.

Despite the important role of ALDHs in plant sterility restoration, and environmental stress responses, only two reported crystal structures of ALDH proteins from *Pisum sativum *have been deposited in the Protein Data Bank (PDB) database up to date. In order to understand the functional mechanism of ALDH2B2/RF2A mediating male sterility restoration and other functions in maize, we analyzed in detail the conformational features of maize ALDH2B2 using computational biology. We obtained the best predicted model of the maize RF2A/ALDH2B2, a mitochondrial associated protein, based on the ten best structural templates and the crystal structures of mitochondrial ALDHs from different organisms deposited in the Protein Database (Figure 3). To better understand the boundary of the catalytic, the cofactor and the oligomerization domains of the protein, we colored coded the corresponding domains, and highlighted the predicted amino acids Cys311 and Glu278, which drive the ALDH reaction with the aldehyde substrate (Figure 3) [16,17]. The quality of the modeled protein was estimated by the C-score values generated by I-TASSER software, which reflects the coverage parameters in the structural simulations and the sequence alignment with the template. C-score is a confidence scoring function to assessing the quality of a prediction and estimate the accuracy of the I-TASSER software predictions, which is based on the quality of the threading alignments and the convergence of I-TASSER's structural assembly refinement simulations. Typically, a good predicted model is obtained when the estimated level of confidence (C-score) is between -5 and 2. The quality of the modeled protein as revealed by the C-score of 1.58 and the percentage identity with the protein template (Table 3) is good, because this value/level of confidence (C-score) ranges between -5 and 2, which is the limit of the acceptable structural model prediction. The level of confidence for all our predicted maize ALDH models were in the range of -0.08 to 1.58 (Table 3), indicating that the protein structures were constructed with high accuracy. Other parameters like TM-score and root mean square deviation (RMSD) were used to check the topology and structural similarity of the models [18]. For ALDH2B2/RF2A, both parameters were scored as 0.94 ± 0.06 and 4.0 ± 2.7Å respectively. TM-score is used to assess the topological similarity of two protein structures, while RMSD is the measure of average distance between the backbones of superimposed proteins. The RMSD values of the predicted models and the templates although highly variable despite significant sequence similarity between them (Table 3) are not unusual. They are indeed in the normal range of accepted RMSD values. These values however, can be drastically reduced if the modelled proteins are made from crystallized maize ALDH structures. Unfortunately, there is no single crystallized maize ALDH protein up-to-date. The accepted models were then made from other organismal ALDH templates as indicated in Table 3. The biological usefulness of the predicted protein models relies on the accuracy of the structural prediction. For example, high-resolution models with RMSD values in the range of 1-3 Å are typically generated by the crystallized model (CM) using close homologous templates. Medium-resolution models, roughly in the RMSD range of 3-7 Å are typically generated from distant homologous templates. Even models with the lowest resolution but still with a correct topology predicted by either *ab initio *approaches or based on weak hits from threading, have a number of useful information including protein domain boundary identification, topology recognition and family/superfamily assignment.

**Table 3:** Structural-dependent modeling parameters for the maize ALDH protein superfamily.

Accession Number	Gene annotation	C-score	TM-Score	N° of decoys	Cluster density	RMSD (Å)	Template (higher Z-score)	PSI-BLAST % Identity with the template	**Norm**. Z-score
AC191038.4	ZmALDH2B2	1.58	0.94 ± 0.06	3000	1.2500	4.0 ± 2.7	1ag8A	53	11.25
AC196114.2	ZmALDH3H1	0.50	0.78 ± 0.10	2356	0.3927	6.2 ± 3.8Å	1ad3A	48	10.84
AC191786.3	ZmALDH5F1	-0.08	0.70 ± 0.12	2056	0.2240	7.6 ± 4.3Å	1ez0A	56	5.67
AC191354.3	ZmALDH6B1	0.43	0.77 ± 0.10	2227	0.3787	6.4 ± 3.9Å	1a4sA	28	7.75
AC196479.3	ZmALDH7B6	1.57	0.93 ± 0.06	2964	1.2350	4.1 ± 2.7Å	1jg7A	61	4.45
AC193454.3	ZmALD10A8	1.20	0.88 ± 0.07	3000	0.7692	4.6 ± 3.0Å	1ad3A	40	12.41
AC219083.3	ZmALD11A3	1.20	0.88 ± 0.07	2996	0.7989	4.8 ± 3.1Å	1bxsA	31	10.36

**Figure 4:**
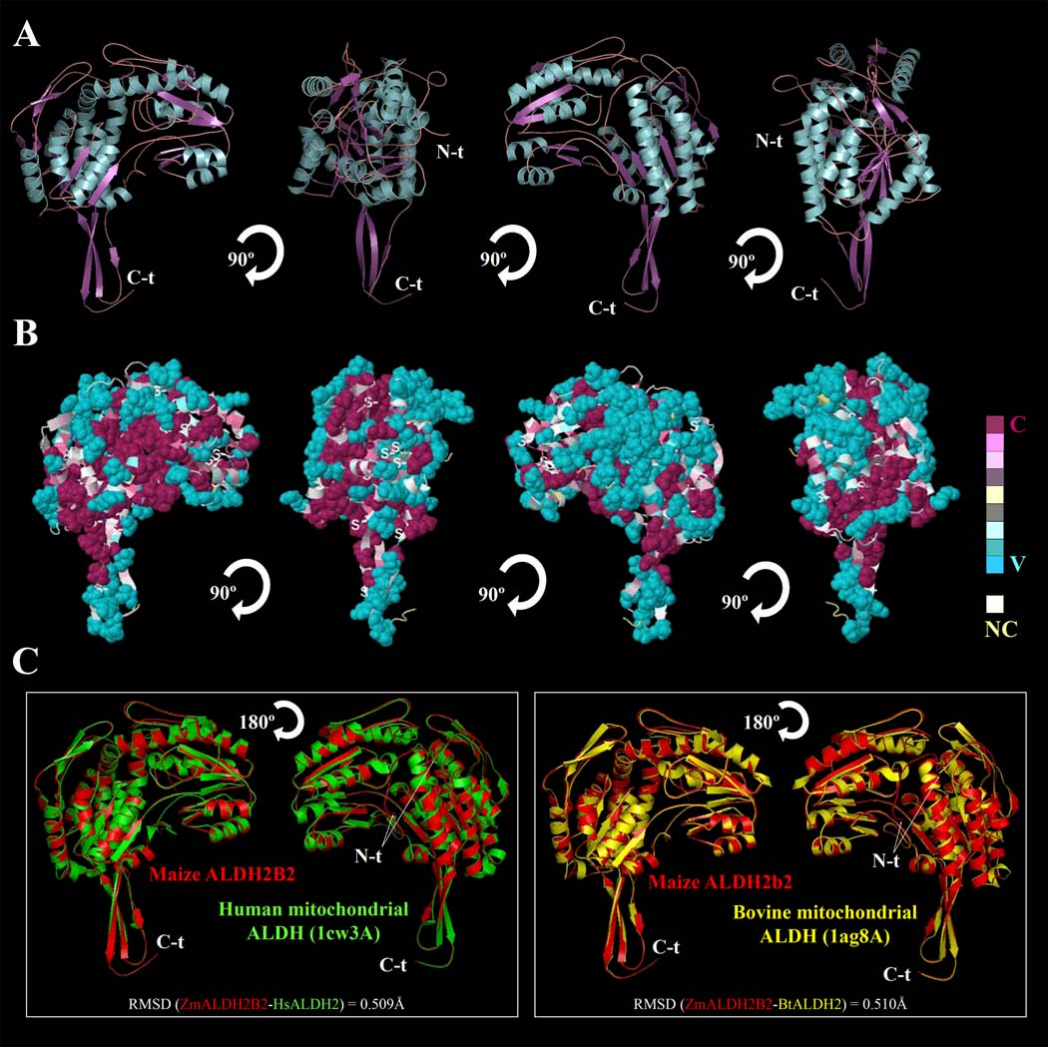
**Structural conformation and conservation analysis of maize ALDH2B2/RF2A**. (A) 3 D structural analysis of the best predicted maize ALDH2B2 model is depicted as a cartoon diagram. Different elements of the secondary structure are colored in blue (α-helix), purple arrows (β-sheet) and purple lines (coils). Each structure is rotated 90° to show different view side of the protein. (B) Conservation profile of the protein using consurf-conservational analysis. The protein was visualized using FirstGlance in Jmol with color-coded conservation scores. The conserved and variable residues are presented as space-filled models and colored according to the conservation scores. (C) Structural comparison of superimposition of ZmALDH2B2/RF2A (red) and human (PDB: 1cw3A) (green) and bovine (PDB: 1ag8A) (yellow) mitochondrial ALDHs. 2 D structural elements comparison, show a small deviations (RMSD) between protein conformations.

The general structure of ALDH2B2/RF2A shows the typical common strands and helices in the Rossmann folding type depicted in different views (Figure 4A). In order to study the specific domain structures, we examined the conservational residue pattern of the surface as well as the active pocket of the protein. The most variable surface residues (depicted in blue) are on the periphery of ALDH2B2/RF2A and the conserved residues (depicted in purple) located in the core of the protein structures (Figure 4B). Generally, residues that are implicated in the biological processes such as protein-protein and protein-ligand interactions are solvent accessible, and residues implicated in protein structure and folding stability are located in the core of the protein. Our findings revealed that maize ALDH2B2/RF2A-coenzyme pocket is highly conserved, while the surface of the opposite side of the pocket is highly variable (Figure 4B).

The structural comparison of maize ALDH2B2/RF2A with other mitochondrial ALDH orthologs allowed us to further validate the accuracy of the modeled maize ALDH2B2/RF2A. We performed a structural superimposition of the maize ALDH2B2/RF2A with crystallized mitochondrial ALDH2B2 from different organisms (human and bovine). The structural protein superimposition (Figure 4C), reveals very little structural deviations (RMSD <0.515Å). However, the noticeable structural differences were located mainly in the tail of the N-term (N-t) domain (Figure 4C). In addition, we observed small differences in some 2 D structural elements (Figure 4C). In summary, the global topology was quite similar to the crystallized proteins, indicating that the modeled ZmALDH2B2/RF2A reflects the crystal-like structure, and represents the most accurate structure of the protein ever reported (Table 3).

**Figure 5:**
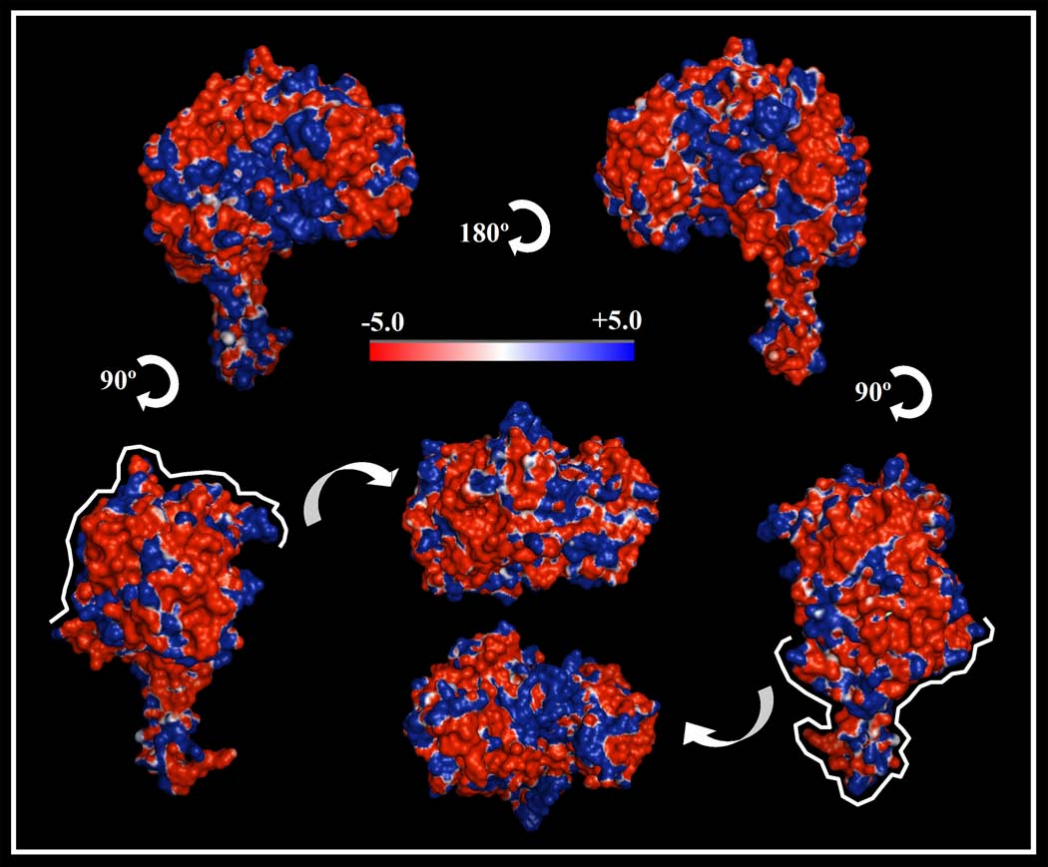
**Electrostatic surface analysis of maize ALDH2B2**. Electrostatic potential (isocontour value of ± 5 kT/e) surface of maize ALDH2B2/RF2A with surface amino acid charges are depicted in red (negative charge) and blue (positive charge). Neutral elements are depicted in white color. Top and bottom views are highlighted with a white line coming from front view.

We next explored and generated the electrostatic surface potentials of maize ALDH2B2/RF2A. We examined the surface charge distribution in this protein using the Adaptive Poisson-Boltzmann Solver (APBS) package [19] as shown in Figure 5. The depicted colors indicate the different surface properties, with red representing negative charge, blue positive and white neutral (Figure 5). To further present a detailed view of ZmALDH2B2/RF2A surface properties, we showed the data in six surface plots/views, which correspond to rotations around the vertical (Z) axis (lateral views; front and back views) and the horizontal (X) axis (top and bottom views) (Figure 5). Overall, the predominant electrostatic potential surface of ZmALDH2B2/RF2A is negative (Figure 5) as indicated by the color coded pattern. However, positively charged amino acids were observed along the surface, and a visible positive region around the cofactor cleft region, and the interface between the coenzyme and catalytic domain are clearly observed (Figure 5).

### Sorting out ZmALDH2B2/RF2A structural features

**Figure 6:**
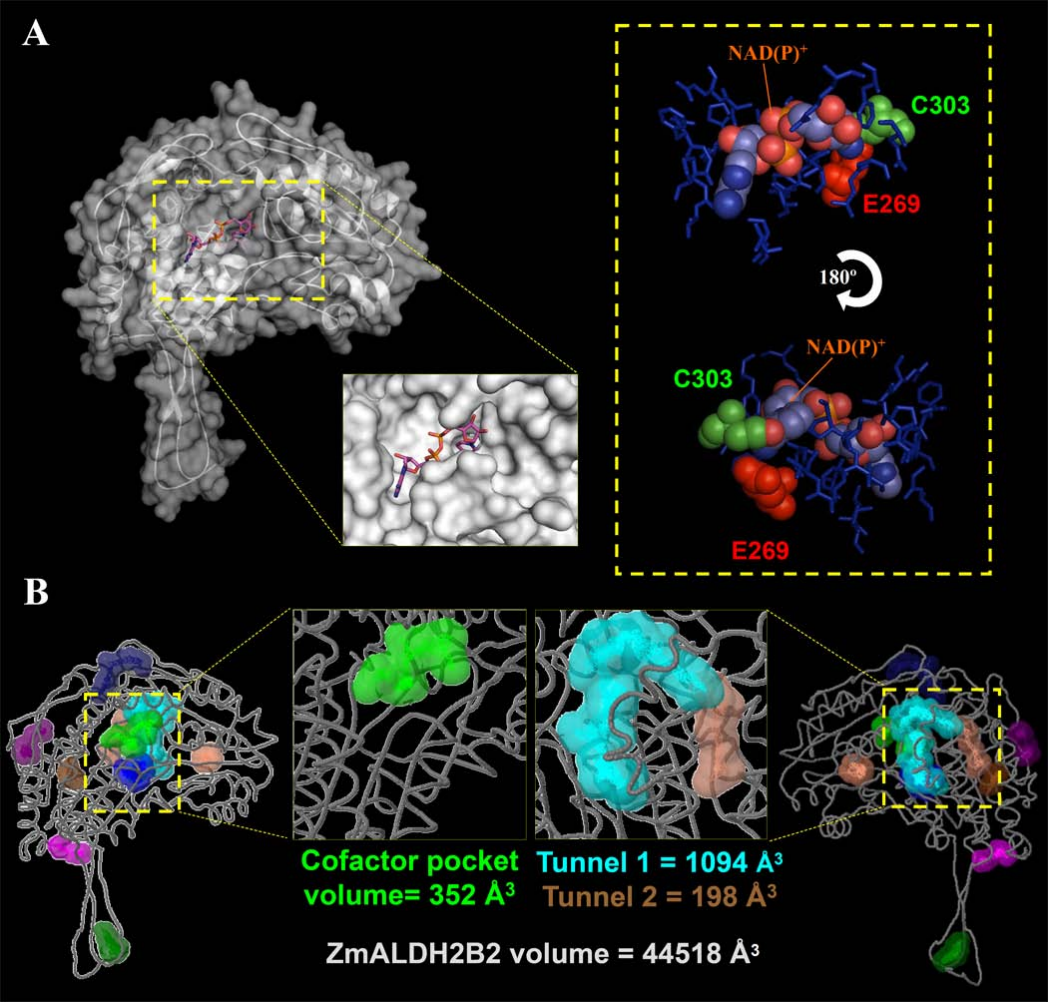
**ZmALDH2B2/RF2A structural surface and pocket/cavity element analysis**. (A) The surface conformation of ZmALDH2B2/RF2A is depicted showing the secondary structure elements inside. The morphology of the cavity accommodating NAD(P)+ cofactor is represented in high magnification. Detail view organization of the predicted amino acids of the pocket is represented in blue color. The space-filled representation of van der Waals surface of the cofactor, and the catalytic amino acid residues Cys 312 (green) and Glu 278 (red) that interact with the aldehyde substrate and drive the MAD(P)^+ ^cofactor dependent reaction is depicted. (B) Cavity and pocket analysis of ZmALDH2B2/RF2A shows nine pockets, and a big tunnel made of two continuous pockets with a total volume of 1292Å^3^. A detailed view of the cofactor binding pocket and the big tunnel in the opposite side of the structure is shown.

Pocket/cavities mapping analysis of ZmALDH2B2/RF2A revealed different interesting features (Figure 6A). For the first time we provide here the anatomy of the catalytic clefts, the ligand-binding pockets and the structural tunnels of ZmALDH2B2/RF2A. As shown in Figure 6(A, B), we detected various hidden specific pockets in ZmALDH2B2. The structural variability of these pockets reflects the multifunctionality features of ZmALDH2B2. The ALDHs have been reported to have variable conformations between non-homologous proteins just like the ligand molecules, but it is also possible that the shapes of different protein binding pockets that bind the same ligand vary [20]. Comparative residue analyses of conserved NADP^+^-dependent binding sites with those of well characterized/crystallized ALDH structures are crucial for the prediction of cofactor specificity and enzymatic mechanism. In well characterized/crystallized ALDHs, there is always a conserved Glu residue (whose position varies according to individual protein sequences) located on the opposite side of another conserved Cys residue at the NAD ring cavity formation. These residues are known to be implicated in proton abstraction from a Cys residue during the ALDH biochemical reaction. Our computational modeling predicted that Glu and Cys residues were respectively positioned at 278 and 312 in the ZmALDH2B2/RF2A primary protein sequence (Figure 6A).

**Figure 7:**
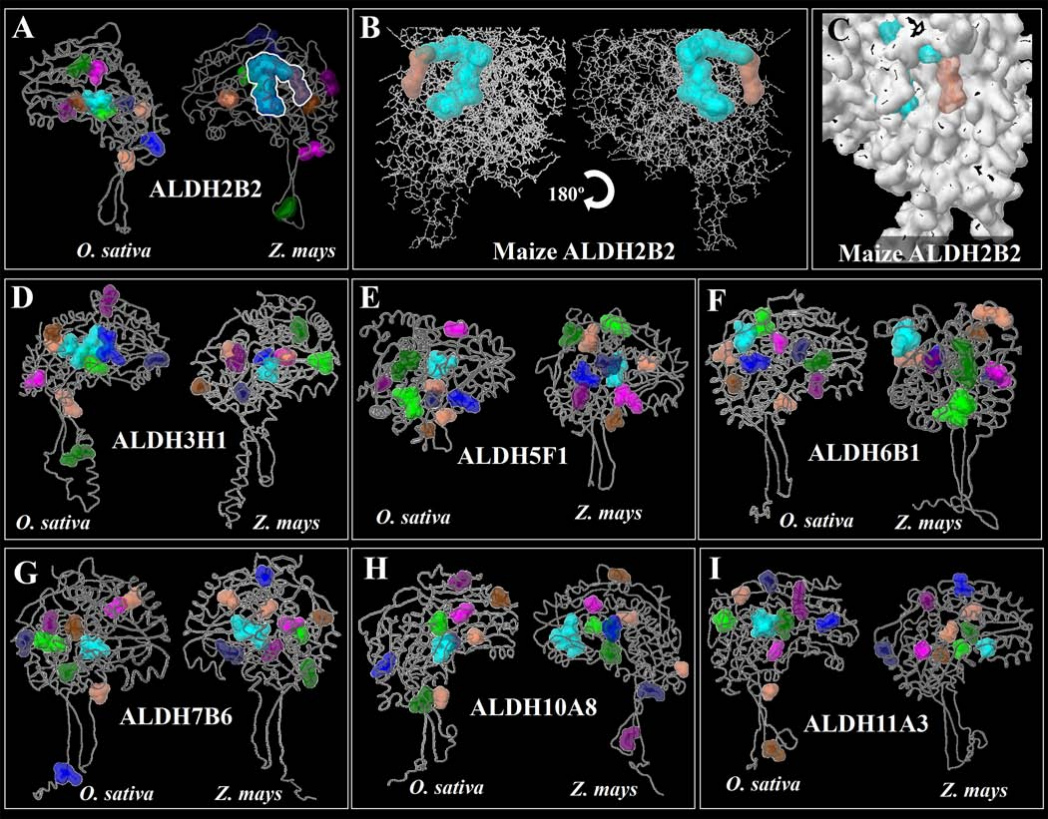
**Pockets and ligand-binding sites comparison**. Prediction and structural analysis of pocket and/or ligand-binding sites were done for selected members of rice and maize ALDH families. The general structure of each ALDH was represented in a stick model and the space-full model was used to depict the pockets and/or ligand-binding sites. An average number of 9 pockets were predicted in each ALDH protein analyzed.

The RF2A protein has a broad substrate spectrum including aliphatic long chain and aromatic aldehydes [7]. mtALDHs typically have many potential substrates [21]. So far, the task of determining the specific aldehyde(s) substrate of RF2A that must be oxidized during fertility restoration is particularly challenging. Biochemical approaches to defining this substrate are complicated by the fact that mutants of the *RF2 *gene exert their effects on male fertility (at least in T cytoplasm maize) in only a single internal cell layer of the anther (i.e., the tapetum). To overcome the limitation of biochemical and genetic approach and verify the ability of RF2A to oxidize a broad substrate spectrum including aliphatic long chain aldehydes, we here used computational biology to address this crucial question. We next sought to uncover some hidden structural features of ZmALDH2B2/RF2A mediating other functions. To do so, we carried out a detailed anatomic analysis of the entire pockets/cavities (with the exception of NAD(P)-binding cavity). We here focused our attention on the geometry of ligand-binding sites to predict and unravel possible hidden ligand binding properties of ZmALDH2B2/RF2A. We first hypothesized that if ZmALDH2B2/RF2A mediating male sterility restoration is dependent on specific protein structural features, these features will only be found in ZmALDH2B2/RF2A, owing to the fact that ZmALDH2B2/RF2A is the only plant ALDH known to play such function. Interestingly, we found that ZmALDH2B2/RF2A has a tunnel-like structure (Figure 6B) made of two continuous cavities, which are big enough to hold various ligands and possibly allows other reactions than aldedehyde dehydrogenase activity. If this tunnel-like structure is critical for male sterility restoration, we expected this structure to be absent in other ALDH protein families that lack this functions. To verify our hypothesis, we analyzed the volume and the interactive properties of the ligand binding regions of pockets/cavities from different members of rice and maize ALDH superfamily (Figure 7). An average of 9 pockets were found in individual ALDH structures analyzed across species (Figure 7A, D, E, F, G, H, I). However, only ZmALDH2B2/RF2A has a very spacious tunnel-like cavity as revealed by its large calculated volume (1292Å^3^) (Figure 6B, Figure 7B, C). In addition, we calculated/predicted and proposed possible ligands that could bind to the described cavities (Figure 7). Our data revealed the uniqueness of the ZmALDH2B2/RF2A tunnel characteristics. The amino acids sequence analysis (Figure 6B, Figure 7B, C) showed that the tunnel is predominantly composed of hydrophobic and neutral amino acids (72%), with only 28% of charged amino acids. We postulate that together with its ALDH activity, RF2A/ZmALDH2B2 is the only maize ALDH candidate that can hold a big molecule/ligand of hydrophobic characteristic in its unique and large tunnel. In summary we here provide direct structural evidence that ZmALDH2B2/RF2A has a specific tunnel-like cavity not found in other ALDHs, through which this protein could bind to various molecular ligands mediating other function.

### Functional relevance of RF2A/ALDH2B2 tunnel like cavity

**Figure 8:**
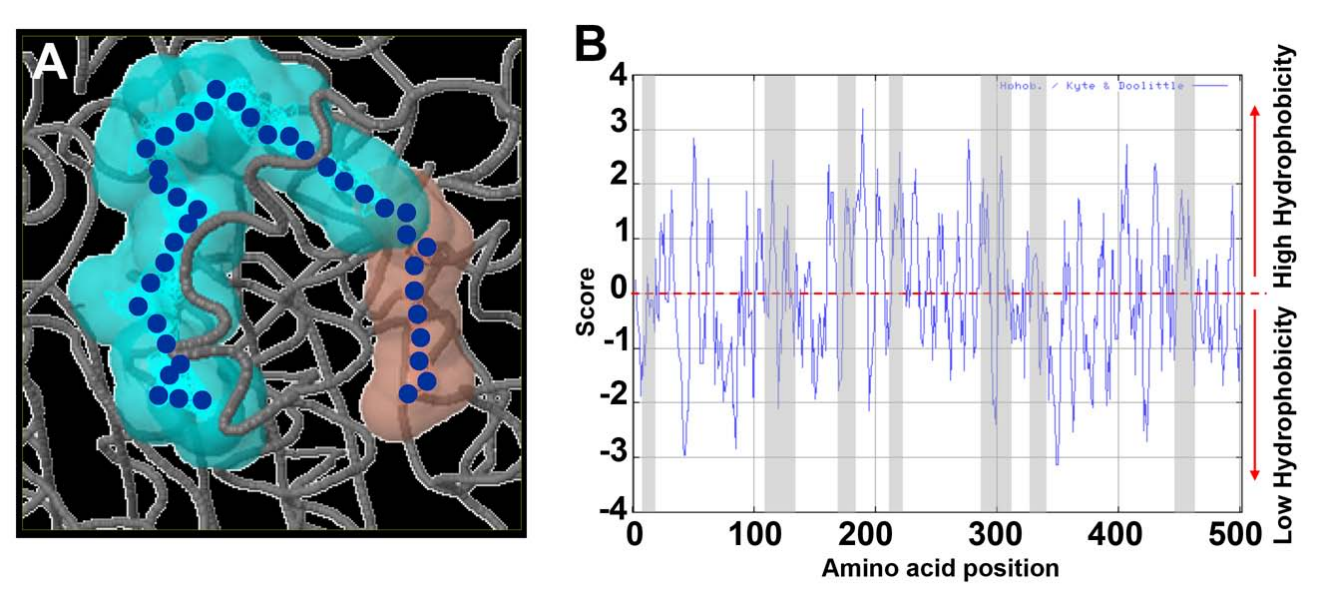
**Structural/physico-chemical properties and hydrophobicity characters of amino acid lining residues of ZmALDH2B2/RF2A tunnel-like cavity**. (A) ZmALDH2B2/RF2A tunnel cavity is depicted to be spacious enough to sequester the 41 carbon chains of the polyketide T-toxin molecule (in blue) or any other molecule of such length, which fits well in the tunnel. (B) Kyte-Doolittle scale for the delineating hydrophobic character of ZmALDH2B2 protein. Regions with values above zero are hydrophobic in character. Shaded regions of the profile represent the residues of the tunnel-like cavity.

The polyketide T-toxin produced by *Cochliobolus heterostrophus *has been shown to bind the plant protein, URF13 causing the formation of pores in the inner membrane of mitochondria [22] and leakage of NAD^+ ^along with other solutes hindering normal mitochondria function [23]. The interaction between URF13 and the polyketide from the fungus leads to southern corn leaf blight disease susceptibility. Due to the spacious volume and the physic-chemical property of RF2A/ALDH2B2 tunnel like cavity, we hypothesized that it might be involved in long chain molecule and or polyketide T-toxin (PKT) sequestration. To test our hypothesis, we compared the physico-chemical properties of RF2A/ALDH2B2 tunnel like cavity with well characterized PKT binding sites in various organisms [24]. The structural models of various iterative PKT domains or sequence stretches that can potentially control the size and extent of unsaturated substrates were then analyzed. In addition, the cavity lining residues (CLRs) and cavity volumes of the active pocket sites were analyzed. This allowed us to correlate the cavity volume and hydrophobicity of the active pocket sites to the number of iterations and the degree of unsaturation of the polyketide products they can hold (Figure 8A). Since T-toxin is a reducing PKS having a greater proportion of saturated carbons [24], we hypothesized that the physico-chemical property of the cavity sequesting T-toxin will be more hydrophobic in order to accommodate the higher proportion of saturated carbon chain of T-toxin molecule. Indeed hydrophobicity cavity lining residues analysis revealed a higher degree of hydrophobicity of the amino acid residues integreting the RF2A/ALDH2B2 tonnel-like cavity structure as expected (Figure 8B). However, polyketides can contain several hydroxyl groups and some times unsaturated double bonds that required some levels of hydrophilic property to chemically fit into the cavity. Consistant to this characteristic, we observed also distinct but relatively suttle region of hydrophilic property certainly required for the accomodation of the carbonyl groups of T-toxin molecule (Figure 8B). It is known that smallest cavities (300Å^3^) belong to the MSAS type PKSs that perform three iterations [24]. Intermediate sized cavities (800Å^3^) belong to the napthopyrone (NAP) like PKSs that iterate from five to eight times [24]. The largest cavities, 1780Å^3^, were observed for the T-Toxin models, which perform 20 iterations with the ligands [24]. As shown in Figure 6B, the RF2A/ALDH2B2 tunnel like cavity falls into the large volume cavity group with its estimated volume of 1292Å^3^. Furthermore, the amino acid residue analysis (Figure 8B) and the physical property of RF2A/ALDH2B2 tunnel cavity correlate perfectly with the characteristic of T-toxin interactive pocket site, suggesting indeed that RF2A/ALD2B2 might be able to bind/hold/sequester the T-toxin or any other toxic molecule as a ligand through its unique tunnel like cavity, by simply trapping the toxin into its big pocket/cavity. However, this interaction will still need to be supported experimentally.

## Discussion

Cellular functions are carried out by 3 D well folded protein structures, protein-protein and potein-ligand interactions. Given that nearly half of the fully sequenced maize genome is yet to be functionally annotated [25], completion of this daunting task is paramount importance in order to elucidate the structural features of individual proteins to gain insights into their functional interaction network. In this study, we identified, annotated, and provided for the first time detailed structural features of selected members of maize ALDH protein families. ALDH proteins play essential roles in metabolic pathways that are critical for development and response to environmental changes [6]. Using the phylogenetic analysis we uncovered the functional and evolutionary relationship of maize *ALDH *protein superfamily with those of rice, Arabidopsis, moss and algae. Although the evolutionary relationships of ALDHs have been the focus of extensive studies [7,14], detailed functional characterization of maize ALDH proteins has never been investigated. The maize genome database contains 24 genes encoding members of 10 *ALDH *gene families (Table 1), which are also represented in other angiosperm plants including rice, poplar and grape [26]. Maize-*ALDH *gene superfamily is the most expanded of plant *ALDHs *ever characterized. A partial explanation for so many maize *ALDH *genes is probably the need to provide ALDH activity in various subcellular compartments. Although some aldehydes (e.g. acetaldehyde) are able to move from one subcellular compartment to another, the molecular sizes of others preclude their passive diffusion across membranes. This probably justifies the presence of multiple organelle-specific ALDHs identified not only in maize (Table 1), but also in rice [15], Arabidopsis [7] and other plant species [8]. The phylogenetic analysis demonstrates that maize and rice ALDHs split up into ten protein families (Figure 1), confirming that these two plant species are indeed monocots. When compared to other plant species, the evolutionary relationships could not be traced to the 10 protein family clades. Instead, they are split into four major clades (Figure 2), revealing some interesting observations; ALDH families 2, 5 and 10 seem to cluster together, suggesting that these families probably diverged from a common ancestor. Finally, the predicted cytosolic and mitochondrial ALDH forms in family 2 can be clearly separated from each other. This is in accordance with results of recently characterized ALDH2 genes from Arabidopsis and rice [7].

Although the Arabidopsis genome sequence has provided a major key for the identification of crucial genes in plants, the functions of grass-specific genes need to be elucidated to gain genetic control of biomass yield, environmental stress response, and quality in food crops [27]. Using computational biology, we attempted in this paper to uncover for the first time some hidden structural features of maize RF2A/ALDH2B2 gene product, a member of family 2 ALDH proteins. Class 2 maize ALDH2B2/RF2A was the first plant ALDH ever characterized [28]. RF2A encodes a nuclear restorer of cytoplasmic male sterility [28,29] and functions in concert with RF1 to restore CMS in maize. Although RF2 proteins have been identified and characterized from various organisms, the mechanistic process of maize RF2A/ALDH2B2 sterility restoration is unknown. The Texas (T) cytoplasm male-sterile (T-CMS) maize had never attracted attention until the occurrence of southern corn leaf blight disease in 1972 [30] caused by a host selective toxin (T-toxin) produced by *Cochliobolus heterostrophus *(race T). T-CMS maize is highly sensitive to T-toxin of *C. heterostrophus *[31]. In T-CMS maize, the genomes of T cytoplasm mitochondria contain a single mitochondrial gene encoding for URF13 protein. URF13 accumulates in the inner membrane of the mitochondria [11,32] causing T-CMS maize to be sensitive to T-toxin. In addition, URF13 severely affects the tapetal cell layer of the anthers, which undergo a premature degeneration at the early microspore stage, resulting in pollen abortion [13]. Genetic and kinetic studies of the maize mitochondrial ALDHs reveal two RF2 proteins (i.e. RF2A and RF2B), and indicate that these two enzymes have similar, but non-identical substrates. The RF2A protein has a broad substrate spectrum including long-chain aliphatic aldehydes and aromatic aldehydes, whereas RF2B can oxidize only short-chain aliphatic aldehydes [33]. Interestingly, these two mitochondrial ALDHs do not accumulate in the same tissues or at the same times [33]. It appears that plant mitochondrial ALDHs have undergone functional specialization. This is confirmed by the observation of specific structural features that distinguish members of mitochondrial ALDHs from each other (Figure 6B, Figure 7). To better understand the functional specialization of mitochondrial maize ALDHs, we analyzed in detail all the structural pockets/cavities of RF2A in comparison with various mitochondrial ALDH proteins from other plant species. Our data revealed distinct structural features of RF2A/ALDH2B2 that might mediate novel ligand binding or other functional specialization. Our structural analysis clearly displayed the uniqueness of the maize ALDH2B2/RF2A tunnel cavity (Figure 6B, Figure 7). This tunnel-like structure can hold up medium and long-chain aliphatic molecules that may be/are harmful to the mitochondria. Amino acid sequence analysis of the cavity revealed that this tunnel is made of neutral and hydrophobic residues suitable for harboring big/long lipophilic and hydrophobic molecules such as the T-toxin (Figure 8B), although this interaction needs to be experimentally tested.

## Conclusion

We have identified for the first time all members of the ALDH protein superfamily in maize; provided a revised, unified nomenclature for these ALDH proteins; analyze the molecular relationship among maize ALDHs compared to other well characterized plant ALDHs. Our computational modeling analysis revealed a spacious tunnel like cavity in RF2A/ALDH2B2, a member of class 2 maize ALDHs, never reported before through which this protein might functionally diverged from other mitochondrial plant ALDHs. Our data suggested that RF2A/ALDH2B2 might interact with long aliphatic chain molecules and other harmful substrates/molecules through its tunnel like cavity to prevent their detrimental effects on mitochondrial organelles.

## Methods

### ALDH sequences search and bioinformatics

Previously identified Arabidopsis- and rice-ALDH sequences retrieved from NCBI http://www.ncbi.nlm.nih.gov/, and rice genomic database (TIGR Rice Annotation Release 4, http://blast.jcvi.org/euk-blast/index.cgi?project=osa1) were used to search for maize ALDH and ALDH-like DNA sequences from the maize genome release 4a.53 http://www.maizesequence.org[9] using BLASTX, BLASTN and BLAST 2.2.24 release (low complexity filter; and based on Blosum62 substitution matrix) [34,35].

Protein motifs of the identified maize-ALDHs were queried using using the PROSITE release 20.66 [36], Pfam 23.0 [37], CDD v2.25 (Conserved Domain Database) or CDART (Conserved Domain Architecture Retrieval Tool) tools [38,39]. After the aboved databases were run, the retrieved sequences were then double checked using Pfam 00171 (ALDH family), PS00070 (ALDH cysteine active site), PS00687 (ALDH glutamic acid active site), KOG2450 (aldehyde dehydrogenase), KOG2451 (aldehyde dehydrogenase), KOG 2453 (aldehyde dehydrogenase) and KOG2456 (aldehyde dehydrogenase) for the identification domains for maize ALDH protein superfamily. Putative functions were thereafter assigned to predicted proteins based upon significant similarity to functionally characterized proteins as priviously described [15].

The maize ALDH deduced polypeptides were then annotated using criteria established by the ALDH Gene Nomenclature Committee (AGNC) [10]. Based on AGNC-annotation criteria, deduced amino acid sequences that are more than 40% identical to other previously identified ALDH sequences compose a family, and sequences more than 60% identical compose a protein subfamily. Deduced amino acid sequences less than 40% identical would describe a new ALDH protein family.

### Sequence alignments and phylogenetic analyses

Sequence alignments of the complete deduced ALDH sequences from *Z. mays*, *O. sativa*, *A. thaliana*, *P. patens *and *C. Reinhardtii *were created in ClustalW v1.81 [40] using the Gonnet protein weight matrix, multiple alignment gap opening/extension penalties of 10/0.5 and pairwise gap opening/extension penalties of 10/0.1. These alignments were adjusted using Bioedit V7.0.5.3 [41]. The unreliable portions of the sequence in the alignment were eliminated. Phylogenetic trees were generated by neighbor-joining (NJ). The estimation of the phylogeny topology of the branches was tested with 1000 bootstrap replicates using the neighbor-joining method. Maize and rice tree was visualized with Treeview v.0.5.0 [42] and the more expanded tree composed of *Z. mays*, *O. sativa*, *A. thaliana*, *P. patens *and *C. Reinhardtii *ALDHs was visualized with Treedyn 198.3 [43].

### Protein modeling, molecular conservation and structural analysis

To better understand the molecular mechanism of ALDH2B2/RF2A mediated male fertility restoration in cms-T, the deduced ZmALDH2B2 protein sequence was modelled using the top 10 PDB closed templates structures by I-Tasser [44]. An initial structural model was generated, and subjected to an energy minimization procedure with GROMOS96 [45], implemented in DeepView/Swiss-PDB Viewer v3.7 [46] to reduce poor van der Waals contacts and correct the stereochemistry of the model. For each sequence analyzed, the quality of the model produced was assessed by checking the protein sterology using the PROCHECK v.3.5 [47] and the energy was checked by ANOLEA [48]. The Ramachandran statistic plots were checked and main numbers of amino acid residues in favorable regions were shown for all the models.

The predicted organic binding site was based on the identification of analogs with similar binding sites taking into account their BS-scores, TM-scores (a scale for measuring the structural similarity between two structures), IDEN (percentage sequence identity in the structurally aligned region), the coverage of the alignment by TM-align, the COV of the model, and the structural alignment (which is equal to the number of structurally aligned residues divided by their length). A BS-score value of > 0.5 signifies a binding site prediction with high confidence. The ligand(s) in the analog structure were then transferred onto the model and the fitness of the ligand-model complex (BS-score) was calculated by comparing the local structure and sequence similarity in the binding site region.

The ConSurf conservation analysis (ConSurf v3.0) [49] was made by evolutionary related conservation scores of the residues for functional region identification from proteins of known three dimensional structures. The degree of conservation of the amino-acid sites among 50 close sequence homologues (Identification of functional regions by surface-mapping of phylogenetic information) was estimated. The conservation grades were projected onto the molecular surface of the proteins to reveal the patches of highly conserved residues that are often important for biological function.

Electrostatic Poisson-Boltzmann (PB) potentials were obtained using APBS v1.2.0 [19] molecular modeling software PyMol 0.99 (DeLano Scientific LLC) with ff99 forcefield of AMBER package [50] to assign the charges and radii to all of the atoms (including hydrogens), which were added and optimized with PDB 2PQR[51], a Python software package that automates many of the common tasks used to prepare structures for continuum electrostatics calculations and provides a platform-independent tool for converting protein files in PDB format to PQR format. Fine grid spacing of 0.35 Å was used to solve the linearized PB equation in sequential-focusing multigrid calculations in a mesh of 161 points per dimension at 300.00 K. Dielectric constants were 2 for the protein and 80.00 for water. The output mesh was processed in scalar OpenDX format to map the PB onto the surfaces with PyMOL 0.99. Potential values are given in units of kT per unit charge (k, Boltzmann's constant; T, temperature).

Pockets/cavities and tunnels were obtained using the interaction energy between the protein and a van der Waals probe. Favorable binding sites were located energetically, and clustered according to their spatial proximity, to be ranked according to the sum of interaction energies for sites within each cluster [52].

Protein-ligand interaction sites prediction were calculated by binding hydrophobic (CH3) probes to the protein, and finding clusters of probes with the most favorable binding energy [52]. To understand the physic-chemical characteristic of the amino acids integrating the tunnel-like cavity in ZmALDH2B2, the hydrophobicities of amino acid sequence were plotted using the kyte-Doolittle hydropathy prediction algorithm [53] by using ProtScale, one of the tools located in the ExPASy Proteomics Server. For this prediction a a window size of 5 was used, and the region/domain of the tunnel-like cavity are highlight in gray.

## Authors' contributions

SOK conceived and designed the experiments. JCJL, EWG performed the experiments. EWG, MJS, JCJL, SOK analyzed the data. MJS, SOK contributed reagents/materials/analysis tools. SOK, EWG, MJS wrote the paper. All authors have read and approved the final manuscript.
